# Efficacy and Ease of Use of a Newly Designed Pencil-Point Epidural Needle Compared to Conventional Tuohy Epidural Needle: A Randomized Single-Blind Pilot Study

**DOI:** 10.7759/cureus.30473

**Published:** 2022-10-19

**Authors:** Fouad G Souki, Reine Zbeidy

**Affiliations:** 1 Anesthesiology, University of Miami Miller School of Medicine, Jackson Memorial Hospital, Miami, USA

**Keywords:** combined spinal epidural, dural puncture, obstetrics, neuraxial anesthesia, pencil point needle

## Abstract

Background and objective

Accidental dural puncture (ADP) and consequent post-dural puncture headache (PDPH) related to epidural needle use have prompted the design of a pencil-point epidural needle. The aim of this prospective, randomized, single-blind pilot study was to assess the efficacy, ease of use, patient satisfaction, and adverse events associated with this newly designed pencil-point epidural needle compared to a Tuohy conventional epidural needle in parturients receiving combined spinal-epidural (CSE) anesthesia for labor.

Methods

After obtaining the Institutional Research Board approval, 100 parturients were randomized to receive CSE anesthesia with either the new pencil-point epidural needle (Gertie Marx, IMD Inc., Huntsville, UT) (P group) or Tuohy needle (T group). We documented patients’ height, weight, loss of resistance (LOR), number of attempts required, onset time of spinal anesthesia, difficulties with insertion of spinal needle, difficulties with insertion of the epidural needle and catheter, duration of the procedure, overall satisfaction of the provider and patient, ADP, PDPH, paresthesia, and pain.

Results

There was no difference in body mass index (BMI), LOR, number of attempts, and onset time of spinal anesthetic between the study groups. Success in obtaining cerebrospinal fluid (CSF) on the first attempt was 50/51 (98%) in the T group vs. 44/49 (89.8%) in the P group (p=0.108). The need for subsequent epidural needle readjustment to obtain CSF was higher in the P group (16/49, 32.7%) vs. the T group (3/51, 5.9%, p<0.001). Success on the first attempt with epidural catheter threading was lower with the pencil-point epidural needle compared to the Tuohy needle (69% vs. 98%, p<0.001). The anesthesiologist switched from the assigned pencil-point epidural needle to the Tuohy needle due to technical difficulties in 8/49 (16.3%) cases. The duration of the procedure was longer in the P group (16.43 ±6.33 minutes) compared to the T group (11.49 ±1.87 minutes) (p<0.001). User satisfaction was lower in the P group compared to the T group (34.7% vs. 90.2%, p<0.001). Patient satisfaction was lower with the pencil-point epidural needle compared to the Tuohy needle (75.5% vs. 92.2%, p*=*0.03). There was no difference in complication rates from the CSE procedure between groups (pain, paresthesia, ADP, and PDPH).

Conclusion

In this pilot study, the use of the pencil-point epidural needle for CSE was associated with less successful epidural catheter placement as well as low user and patient satisfaction compared to the Tuohy epidural needle. Modifications in the pencil-point epidural needle design are needed to improve efficacy and enhance user acceptance before a larger study can be conducted to evaluate the rates of ADP and PDPH.

## Introduction

Neuraxial anesthesia is an integral part of anesthesia practice today. Present methods reflect the achievements of a long process of innovation, mainly in response to the limitations and complications of earlier techniques. Needle design was significant among these improvements [1]. However, the search continues for better instruments and aids that facilitate neuraxial anesthesia and increase patient safety.

The combined spinal-epidural (CSE) technique is frequently used for labor analgesia and anesthesia. The technique entails the placement of the tip of the epidural needle in the epidural space and the insertion of a spinal needle through the epidural needle until it punctures the dura. This is followed by intrathecal injection of medication and epidural catheter placement [2,3]. Although CSE is popular, it is not without side effects, such as difficulty in inserting the spinal needle, inability to thread the epidural catheter, paresthesia, pain, accidental dural puncture (ADP), and post-dural puncture headache (PDPH) [4]. PDPH is a recognized complication of epidural needle use with an incidence of 1-3% in obstetric patients [5]. The incidence of ADP with a Tuohy epidural needle in parturients varies between 0.04% and 6% [6]. PDPH may occur in up to 80% of patients with ADP and is often debilitating [7-11].

There has been scarce research examining the effect of epidural needle design on the incidence of ADP and PDPH. However, improvement in spinal needle design with non-cutting tips has been shown to decrease the incidence of PDPH [12-13]. Based on the design of the non-cutting Sprotte spinal needle, a 19.5-gauge Special Sprotte™ epidural needle with a solid bullet-shaped tip and a lateral opening for passage of an epidural catheter was devised in the past [13]. Experiments using an 18-gauge Special Sprotte™ epidural needle have shown less cerebrospinal fluid (CSF) leakage and PDPH after ADP when compared to a 17-gauge Tuohy needle [13-15]. However, the researchers have concluded that user acceptance of the Special Sprotte™ epidural needle must be considered in further studies [13]. Based on these findings, a new 17-gauge pencil-point epidural needle (Gertie Marx, IMD Inc., Huntsville, UT) has been designed with a rounded pencil-point tip that lacks cutting edges and has a lateral opening for passage of an epidural catheter or spinal needle.

The primary aim of this prospective, randomized, single-blind pilot study was to assess the efficacy and ease of use of the newly designed pencil-point epidural needle compared to the conventional Tuohy epidural needle in parturients receiving CSE anesthesia for labor. The secondary objectives of the study included the evaluation of adverse events and patient satisfaction.

## Materials and methods

After obtaining the Institutional Research Board/Human Subject Research Office approval (#20080532) and receiving written informed consent, a convenient sample of 100 nulliparous or multiparous parturients requesting neuraxial analgesia for labor were recruited to participate in a prospective, randomized, single-blind pilot study at our tertiary care obstetric unit. All women requesting neuraxial analgesia were deemed eligible for recruitment if they were over 18 years of age with an American Society of Anesthesiologists (ASA) physical status (PS) II-III. Patients with recognized contraindications to neuraxial anesthesia, body mass index (BMI) >40 kg/m^2^, history of neurological, neuromuscular, or psychiatric disorders, history of drug abuse, emergent or stat cesarean section, those who were unable to give informed consent and/or comply with study protocol were excluded. A BMI <40 kg/m^2 ^was chosen for our study population as a benchmark since morbidly obese parturients (BMI ≥40 kg/m^2^) experience higher levels of overall anesthesia complications and more complicated placement of regional anesthesia [16].

**Figure 1:**
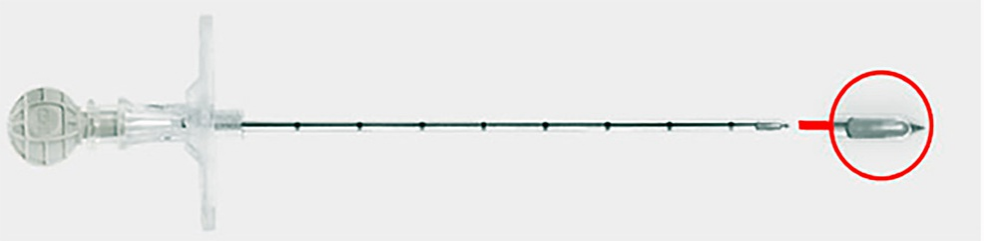
17-gauge pencil-point epidural needle (Gertie Marx, IMD Inc., Huntsville, UT)

At the time of anesthesia request, patients were randomized by computer-generated code to receive either CSE using a 3.5-inch, 17-gauge pencil-point epidural needle (Gertie Marx, IMD Inc.) (Figure 1) (P group) or the conventional 3.5-inch, 17-gauge Tuohy needle (T group). In both groups, a 5-inch, 27-gauge Gertie Marx spinal needle was inserted through the epidural needle. Neuraxial blocks were performed by an obstetric anesthesia fellow or attending anesthesiologist. Parameters monitored included maternal noninvasive arterial blood pressure, pulse oximetry, and fetal heart rate.

CSE was performed via a midline approach between L3-L4 or L4-L5 interspaces in the sitting position using saline for the loss of resistance (LOR) technique with the bevel of the epidural needle facing cephalad. When the free flow of CSF was noted at the hub of the spinal needle, bupivacaine 1.25 milligrams and fentanyl 15 micrograms were injected. If no CSF was obtained, a second attempt at spinal needle insertion was made after redirecting the epidural needle. The number of attempts required to identify the epidural space and the number of needle readjustments to find CSF were recorded.

All parturients had a 19-gauge Arrow Flex Tip Plus (Teleflex, Morrisville, NC) open-end tip catheter placed 4 cm into the epidural space. The catheter was aspirated to check for the presence of blood or CSF and was secured with an occlusive dressing. If the epidural needle accidentally punctured the dura, the catheter was advanced, and a continuous spinal technique was used for labor.

Patients' age, ASA PS, height, weight, the distance of the epidural space from the skin, number of attempts, difficulties with insertion of spinal needle, onset time of spinal anesthesia, difficulty upon insertion of the epidural needle and catheter, paresthesia, pain, unintentional dural puncture with the epidural needle, intrathecal or intravascular catheter placement, and the duration of the procedure were recorded. The duration of the procedure was defined as the start of local infiltration until the removal of the epidural needle after catheter insertion. If technical difficulties were encountered with the initially assigned needle (failure to get CSF through spinal needle or thread epidural catheter after multiple attempts), then the anesthesiologist used the epidural needle of his/her choice. The use of an epidural needle other than the one assigned was also recorded. User satisfaction and patient satisfaction with the CSE placement were recorded using a verbal numeric satisfaction scale of 0-10 (10: completely satisfied, 0: completely unsatisfied).

Patients were informed by the anesthesiologist if an ADP was suspected but the type of needle used was not revealed. All patients were asked about symptoms of PDPH for six consecutive days postoperatively, and whether they experienced ADP or not, by an anesthesia resident blinded to group assignment.

Dichotomous data were compared using Chi-square analysis and Fisher’s exact test where appropriate. Unpaired Student’s t-test was used to compare needles for the number of attempts, patient demographics, duration of the procedure, LOR, and time to onset of neuraxial block. A p-value <0.05 was considered statistically significant.

## Results

A total of 100 parturients were recruited; 51 were assigned to the Tuohy (T) epidural needle group and 49 to the pencil-point (P) epidural needle group (Gertie Marx, IMD Inc.) as per computer-based simple randomization. The two study groups were similar with regard to weight, height, BMI, and level of anesthesiologist training (Table 1).

**Table 1:** Baseline characteristics SD: standard deviation; BMI: body mass index

Variables	Pencil-point (n=49), mean ±SD	Tuohy (n=51), mean ±SD	P-value
Age (years)	27.88 ±6.17	32.55 ±7.28	0.001
Weight (kg)	81.22 ±7.56	81.90 ±14.10	0.762
Height (cm)	1.64 ±0.06	1.63 ±0.06	0.235
BMI (kg/m^2^)	30.25 ±2.71	31.02 ±5.71	0.350

All patients in the P group required a 16-gauge cutting-edge needle to open the skin prior to epidural needle insertion. Groups did not differ with respect to the level of epidural needle placement, patient position during epidural needle insertion, LOR method, number of attempts, depth of the epidural space, and onset time of the block following the intrathecal administration of medication (Table 2). Duration of the procedure (minutes) was higher in the P group (16.43 ±6.33) compared to the T group (11.49 ±1.87) (p<0.001).

**Table 2:** Technical variables SD: standard deviation

Variables	Pencil-point (n=49)	Tuohy (n=51)	P-value
Sitting position, n	49	51	
Loss of resistance to saline, n	49	51	
Loss of resistance (cm), mean ±SD	5.71 ±0.98	5.50 ±0.98	0.278
Number of attempts, mean ±SD	1.51 ±0.68	1.29 ±0.64	0.105
Duration of procedure (minutes), mean ±SD	16.43 ±6.33	11.49 ±1.87	<0.001
Onset time of spinal anesthesia (minutes), mean ±SD	5.59 ±1.22	6.02 ±1.87	0.178

Treatment outcomes are summarized in Table 3. Success in obtaining CSF on the first attempt was 50/51 (98%) in the T group vs. 44/49 (89.8%) in the P group (p=0.108). The need for subsequent epidural needle readjustment was higher in the P group (16/49, 32.7%) vs. the T group (3/51, 5.9%) (p<0.001). Threading the epidural catheter was less successful on the first attempt in the P group vs. the T group (69% vs. 98%, p<0.001). The anesthesiologist chose to switch from the assigned pencil-point epidural needle to the Tuohy needle due to technical difficulties in 8/49 (16.3%) of the cases. No patient assigned to the Tuohy epidural needle was switched to the pencil-point epidural needle.

User satisfaction was lower in the P group (17/49, 34.7%) compared to the T group (46/51, 90.2%) (p<0.001). Patient satisfaction was also lower in the P group (37/49, 75.5%) compared to the T group (47/51, 92.2%) (p=0.03).

Pain upon needle insertion occurred in 9/49 (18.4%) patients in the P group compared to 3/51 (5.9%) in the T group (p=0.069). The incidence of paresthesia during catheter insertion was similar between groups. No ADP or PDPH were noted in either group.

**Table 3:** Treatment outcomes CSF: cerebrospinal fluid; ADP: accidental dural puncture; PDPH: post-dural puncture headache

Outcomes	Pencil-point (n=49)	Tuohy (n=51)	P-value
Skin puncture	0%	100%	<0.001
Success in obtaining CSF on the first attempt	89.8% (44)	98% (50)	0.108
Epidural needle readjustment	32.7% (16)	5.9% (3)	0.001
Success of epidural catheter on the first attempt	69.4% (34)	98% (50)	<0.001
Switched needle	16.3% (8)	0%	0.002
User satisfaction	34.7% (17)	90.2% (46)	<0.001
Patient satisfaction	75.5% (37)	92.2% (47)	0.03
Pain (epidural needle insertion)	18.4% (9)	5.9% (3)	0.069
Paresthesia catheter	4.1% (2)	7.8% (4)	0.678
ADP	0	0	
PDPH	0	0	

## Discussion

While pencil-point non-cutting epidural needles have been evaluated on a few occasions for their potential to reduce PDPH and CSF leak with ADP, their efficacy, ease of use, and patient satisfaction have not been well studied [13-15]. Our work showed that efficacy, ease of use, and patient satisfaction were significantly decreased with the pencil-point epidural needle compared to the Tuohy needle due to several reasons. Firstly, penetration through the skin was unfeasible due to the inherent non-cutting pencil-point design of the epidural needle and had to be facilitated by using a 16-gauge cutting-edge needle to open the skin. Second, the pencil-point epidural needle had to be advanced intermittently because the conical tip could not cut through tissue. A similar concern was reported in a study conducted with the bullet-shaped Special Sprotte^TM^ epidural needle where 36.8% of anesthesiologists described LOR to air as excellent compared to 66.6% with the Tuohy needle [13]. Third, the pencil-point epidural needle had to be readjusted frequently or replaced by the Tuohy needle after the failure to obtain CSF on the first attempt. Fourth, the threading of the epidural catheter through the pencil-point epidural needle was more cumbersome. This can be due to the catheter abutting against the closed tip of the pencil-point epidural needle, or incorrect placement [2,17]. On several occasions, the catheter became lodged in the opening, requiring readjustment of the epidural needle, and in some cases, switching to the Tuohy needle. Fifth, the procedure took longer in the pencil-point epidural group. Consequently, all these shortcomings led to the decreased user and patient satisfaction when the pencil-point epidural needle was used.

Paresthesia is common during the placement of the epidural catheter, with an incidence that varies from 6% to over 50% depending on the level of epidural needle placement, needle orientation, and type of epidural catheter [18-23]. One of the secondary objectives of this study was to determine if there was a difference between the pencil-point and Tuohy epidural needle groups in the occurrence of adverse events, particularly paresthesia. Although the data did not reveal a difference in the occurrence of paresthesia upon catheter insertion between groups, the incidence of paresthesia was low in the pencil-point and Tuohy study groups (4.1 vs. 7.8% respectively, p=0.678), which is in line with studies related to the use of soft-tipped Arrow Flex Tip Plus (Teleflex) epidural catheters [21].

The current study had some limitations that merit discussion. First, the provider’s lack of experience with the pencil-point epidural needle may have contributed to the lower user and patient satisfaction scores. There is a learning curve in developing familiarity with the more subtle LOR of the pencil-point epidural needle [13]. It is possible that with adequate training, the time to complete the procedure with the pencil-point epidural needle would have decreased. Nevertheless, experience with this pencil-point epidural needle could not compensate for the lack of its cutting ability and difficulties in threading the epidural catheter that led practitioners to switch from the pencil-point to the Tuohy epidural needle. Second, the study was not powered enough to detect a difference in ADP and PDPH between groups, and no ADP or PDPH were observed either. The low incidence of ADP and PDPH would have required that 1000 patients be enrolled in each group to detect a reduction in PDPH from 3% to 1% with an alpha of 0.05 and power of 90%.

## Conclusions

ADP and consequent PDPH related to epidural needle use have prompted the design of a pencil-point epidural needle. In this pilot study, the use of the pencil-point epidural needle for CSE was associated with less successful epidural catheter placement as well as low user and patient satisfaction compared to the Tuohy epidural needle. Modifications in pencil-point epidural needle design are needed to improve efficacy and enhance user acceptance before a larger study can be conducted to evaluate the rates of ADP and PDPH.
